# Short-Lived Active Prorenin: Precursor of So-Called Native Prorenin

**DOI:** 10.1161/HYPERTENSIONAHA.124.21368

**Published:** 2024-05-16

**Authors:** Maarten A.D.H. Schalekamp, Jaap Deinum, A.H. Jan Danser

**Affiliations:** 1 Department of Internal Medicine, Erasmus MC, Rotterdam, The Netherlands (M.A.D.H.S., A.H.J.D.).; 2 Department of Internal Medicine, Radboud University MC, Nijmegen, The Netherlands (J.D.).

**Keywords:** angiotensins, antibodies, monoclonal, extracellular fluid, kidney, renin

## Abstract

The enzymatic activity of the aspartic protease, renin, is critical for its function in blood pressure regulation and sodium homeostasis. Incubation of so-called native prorenin at low pH leads to its activation. After binding to transition-state mimicking renin inhibitors at neutral pH, prorenin attains the active conformation, as indicated by immunosorbent assay using monoclonal antibodies specific for epitopes of the prosegment or the renin body. A comparison of immunosorbent assay with enzyme-kinetic assay revealed the intermediary steps of prorenin auto-activation/inactivation. The kinetically identified intermediary steps of activation/inactivation correspond with the published crystal structures of free renin, free prorenin, and renin in complex with inhibitors. Both renin and activated prorenin exist in 2 forms, α and β. The α form is active, and the α/β quantity ratio is 2.5. The kidney produces renin and prorenin, while the ovarium, placenta, and eye produce inactive prorenin. The production of renin by these organs has never been demonstrated. We propose that the so-called native prorenin in extracellular fluid, including the circulation, is derived, at least partly, from short-lived active prorenin. Its potential paracrine function is discussed.

The enzymatic activity of the aspartic protease, renin, is key to the functioning of the renin-angiotensin system. Consensus says that inactive prorenin is the precursor of renin and that in vivo activation of prorenin depends on cleavage of the prosegment from the renin body. Gastric aspartic proteases like pepsinogen are activated at low pH. The early phase of activation is reversible and is followed by proteolytic auto-activation. Prorenin is also activated by acid, but here activation is rapidly reversed at pH 7 and 37 °C. Unlike pepsin, renin is active at neutral pH, and the same is true for acid-activated prorenin.^1,2^

The kidney produces renin and prorenin. Other organs, like the ovary, uterus, and eye, produce prorenin, but there is no evidence of prorenin-to-renin conversion in these organs. Low-molecular weight transition-state-mimicking renin inhibitors interact with native prorenin at neutral pH, thereby inducing the unfolding of the prosegment and a change of the renin moiety into a conformation identical or very similar to native renin. The x-ray crystal structures of free renin, free prorenin, and renin in complex with inhibitors are now available. The pathway of reversible prorenin auto-activation/inactivation is still not fully understood. A better understanding of this process may help to design experiments addressing the question of how prorenin could participate in the functioning of the so-called tissue renin-angiotensin system.

## COMPARISON OF ENZYME-KINETIC ASSAY AND IMMUNOSORBENT ASSAY RESULTS REVEALS AN ACTIVITY ON/OFF SWITCH IN RENIN AND REVERSIBLY ACTIVATED PRORENIN

To compare the results of enzyme-kinetic assay and immunosorbent assay (ISA), both assays have been calibrated against the internationally accepted standard of human kidney renin.^3,4^ In the ISAs reported here, highly purified recombinant human renin (Ciba-Geigy) was used as a standard. The MRC unit is based on the Goldblatt unit; 1 Goldblatt unit is the quantity of renin that, after IV injection, raises arterial blood pressure by 30 mm Hg in a nonanaesthesized trained dog. The Ciba-Geigy standard contains 700 Goldblatt units per mg of protein.^3–5^ Characteristics of various types of ISA are given in Table 1. In Table 2, the results of ISA are compared with those of enzyme-kinetic assay. The results of enzyme-kinetic assay and ISA(R) are not different. The same is true for acid-activated prorenin and for prorenin preincubated with the renin inhibitor, remikiren.^6–8^ The results of ISA(R,PR) are higher with a factor of 1.4. Incubation of native prorenin with the inhibitor VTP-27999 does not induce reactivity to mAb(R). The addition of VTP-27999 to renin or to acid-activated prorenin, however, raises the binding to mAb(R) by a factor of 1.4.^7^

**Table 1.**
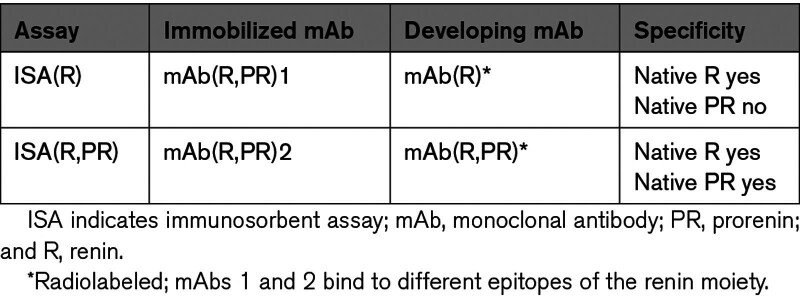
Characteristics of Immunosorbent Assays

**Table 2.**
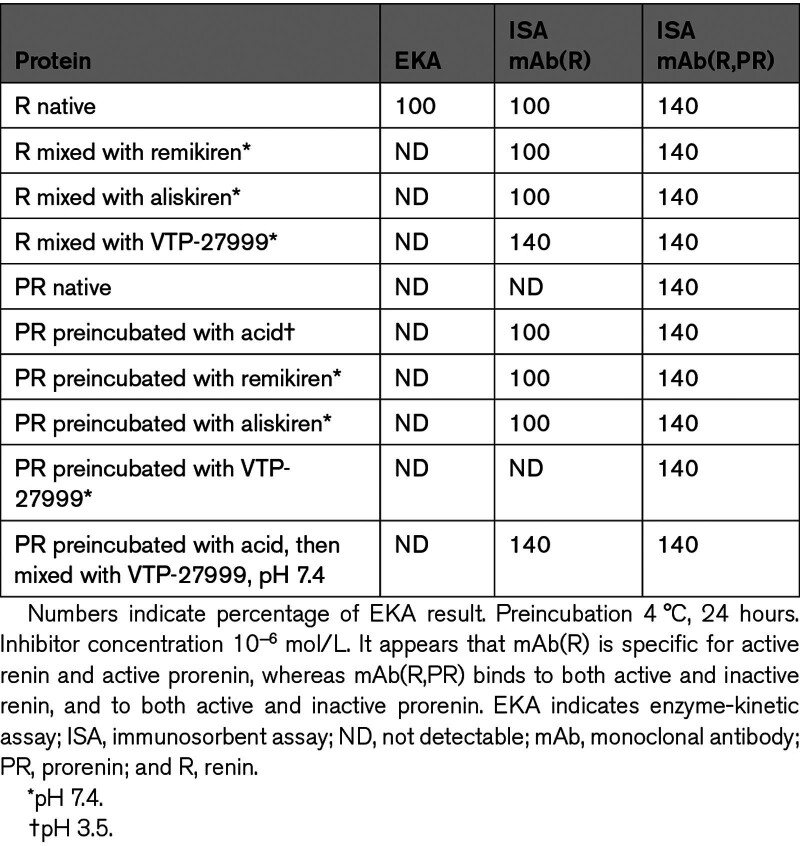
Comparison of EKA and ISA Results

These results demonstrate the presence of 2 forms of renin and reversibly activated prorenin, α and β, with an α/β quantity ratio of 2.5. The α-form has enzymatic activity. Remikiren has similar affinity to the α- and β-forms. VTP-27999 binds selectively to the α-form. These results are in agreement with the fact that the MRC unit is a measure of the quantity of active renin and that the 0.7 μU MRC standard corresponds with 1 pg of renin protein.

The turnover rate of human renin when reacting with human angiotensinogen at physiological pH and temperature is about 1 per second.^4,9^ This low number is probably determined by the time it takes to complete the activity on/off, α/β cycle.

## AUTO-ACTIVATION/INACTIVATION OF PRORENIN: INTERMEDIARY STEPS

Renin belongs to the A1 family of aspartic proteases. Like pepsin, renin has 2 Asp residues in the active site. The general structure of these enzymes consists of 2 similarly folded domains. The active site is situated within the substrate-binding cleft between the 2 lobes: 1 active site Asp belongs to the N-terminal domain and the other to the C-terminal domain. In the so-called native prorenin, part of the prosegment is folded over and within the cleft, thereby inhibiting the binding of the substrate. In addition, the N-terminal domain has a mobile segment, the flap, which allows the substrate to attain the proper position for binding to the active site.

Analysis of the kinetics of the activation of prorenin by acid and the activation induced by renin inhibitors has revealed the intermediary steps of auto-activation/inactivation, with the following intermediary steps^4^:


$$\text{PRf}⇄\text{PRui}\overset{{\text{K}}_{1}}{\underset{{\text{k}}_{2}}{⇄}}\text{PRu}\beta ⇄\text{PRu}\alpha$$

PRf is the so-called native form of PR, in which the prosegment is completely folded and prevents the substrate from entering the substrate-binding cleft. In PRu, the prosegment is unfolded. PRuα is active; PRuβ is inactive. Analysis of the effects of acid and of the transition-state mimicking inhibitor remikiren reveals an inactive intermediate, PRui, in which the prosegment is partially unfolded, so that binding of H^+^ ions or inhibitors leads to further unfolding. Results obtained with the inhibitor aliskiren are comparable with those with remikiren. In contrast, the inhibitor VTP-27999 does not induce activation. The transition from PRui to PRuβ and the reverse are the rate-limiting steps for activation and inactivation. The PRuα/PRuβ and PRui/PRf concentration ratios are 2.5 and 0.02, respectively. At pH 7.4, 37 °C, k_1_ for activation is not measurable, and k_2_ for inactivation is 2.82 h^−1^. At pH 7.4, 4 °C, k_1_=0.011 h^−1^, k_2_=0.032 h^−1^.^2,6^ The general folding of the renin moiety of prorenin, including its active site, is very similar to that of renin itself. As mentioned, renin (R) and inhibitor-activated prorenin (PRu) have 2 conformations: Rα and Rβ, and PRuα and PRuβ. The PRuα/PRuβ and Rα/Rβ quantity ratios are equal.

An important feature of the auto-activation/inactivation process is that PRu to PRf conversion, in contrast to the reverse, is highly temperature-dependent. At physiological temperature and pH, PRf to PRu conversion takes days, whereas the reverse is a matter of minutes, so spontaneous activation is not detectable.^2^ The rapid PRu to PRf conversion makes it difficult to demonstrate the in vivo existence of PRu.

The x-ray crystal structures of renin and the renin part of prorenin have shown that the general folding pattern of the 2 is the same.^10^ Crystals of renin, either free or in complex with remikiren or aliskiren, contain 2 independent molecules in the asymmetrical unit. Monomer A has the closed conformation, that is, the C-terminal domain has moved toward the N-terminal domain. Monomer B is open (Figure).

**Figure.**
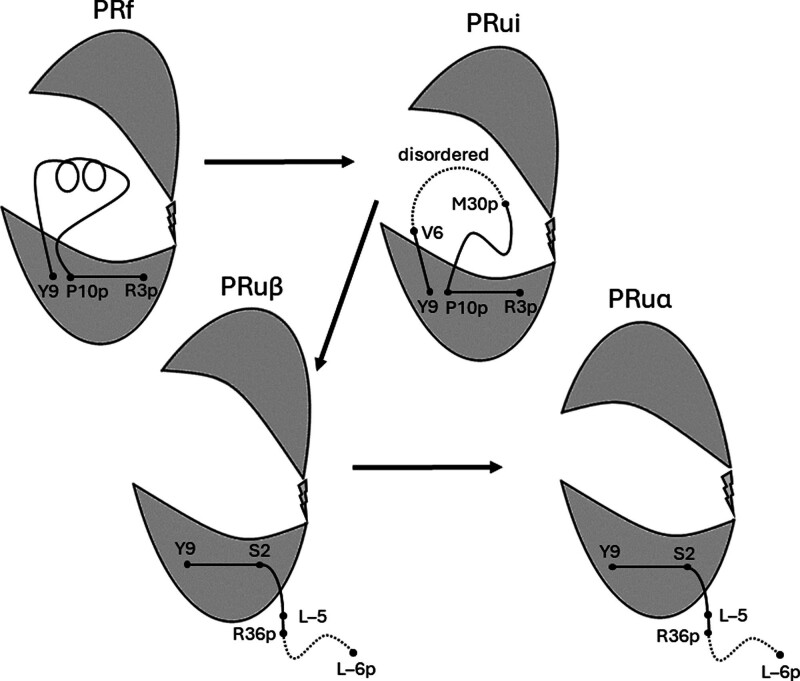
**Cartoon depicting the intermediary steps of prorenin activation/inactivation.** The prosegment (p) is formed by the aminoacids L−6p to R43p, and the renin moiety is formed by L−5 to R326. Some residues of the prosegment and the renin part have a negative sign because of their alignment with pig pepsinogen. The renin part exists in 2 domains, that is, the N-terminal L−5 to I141 domain and the C-terminal Y154 to R326 domain, which are connected by a hinge. In PRf, the prosegment is completely folded, which prevents the entrance of substrate into the cleft between the 2 domains. PRui has the prosegment partially unfolded and disordered, so that the scissile bond between R36p of the prosegment and L−5 of the renin part is exposed to activator proteolytic enzymes, and low-molecular weight inhibitors can reach the active site. In PRu, the prosegment is completely unfolded and outside the substrate-binding cleft. In PRuβ, as in PRf and PRui, the amino acids R3 to P10 of the prosegment form the first strand of the centrally located 6-standed antiparallel β-sheet of prorenin and are in PRu replaced by S2 to Y9.

Crystals of renin in complex with VTPP-27999 also contain monomers A and B, but here they are practically identical and have the closed conformation. Since remikiren and aliskiren have similar affinity to Rα and Rβ, whereas VTP-27999 appears to be specific for Rα,^7^ we propose that Rα is closed and Rβ open, and that the same holds for PRuα and PRuβ.

There are 2 independent molecules in the asymmetrical unit of the prorenin crystal. In the monomers P+A and Q+B, the prosegments are associated with the renin moieties A and B.^11^ The C-terminal halve of Q is disordered and joins B, probably at the outer side of the flap, so that the scissile bond between prosegment and renin is exposed.^11^ This would explain the observation that acid-activated prorenin is about 50× more susceptible to conversion into renin by serine proteases than native prorenin.^4^ It also corresponds with the fact that [PRui]/[PRf]=0.02, and it is possible that PRu, rather than PRf, is the natural precursor of renin. In view of these considerations, we conclude that PRf corresponds with P+A and that PRui corresponds with Q+B.

Prui to PRuβ conversion is the rate-limiting step of the unfolding process, that is, activation. As mentioned, the 3-dimensional structure of PR has 2 domains flanking the substrate-binding cleft. PR and R contain a centrally located 6-stranded antiparallel β-sheet structure; 3 belong to the N-terminal domain, and 3 to the C-terminal domain. The rate-limiting step of activation/inactivation involves the exchange of 1 of the 3 strands of PRf for 1 of the 3 in PRu (Figure). This also occurs when pepsinogen irreversibly changes into pepsin.

## PHYSIOLOGICAL IMPLICATIONS

Increased plasma levels of inactive, intact prorenin, that is, PRf, have been reported in patients affected by diabetes and its microvascular complications. Plasma renin is normal in these patients.^12^ The vitreous contains PRf in concentrations that are higher in diabetics than nondiabetics.^13^ Renin is virtually absent. Concomitant measurements of plasma proteins in vitreous and circulating plasma have indicated that the presence of increased prorenin in vitreous is not caused simply by breakdown of the blood/retinal barrier but by increased production of prorenin in the eye.^13,14^

Very high concentrations of PRf are found in amniotic fluid^15^ and ovarian follicular fluid.^16^ Plasma prorenin is increased in gonadotropin-stimulated women and rises during pregnancy. Observations in a woman with primary ovarian failure showed that plasma prorenin rose during pregnancy, but it remained much lower than in normal pregnant women.^17^ This indicates that normally the ovary is the source.

The half-life of short-lived active, intact prorenin (PRu) is 15 minutes,^2^ which is compatible with a paracrine function. The identification of this active form of prorenin may shed new light on the observation by Luetscher et al^12^, many years ago, that increased plasma prorenin is associated with the presence of diabetic microvascular complications.

The in vitro analysis of the process of nonproteolytic auto-activation/inactivation can help to design experiments that address the existence of active, intact prorenin in vivo and its role in the functioning of local tissue prorenin-angiotensin systems.

## ARTICLE INFORMATION

### Sources of Funding

None.

### Disclosures

None.
